# Feasibility of Using Subject-Collected Dust Samples in Epidemiologic and Clinical Studies of Indoor Allergens

**DOI:** 10.1289/ehp.7648

**Published:** 2005-03-14

**Authors:** Samuel J. Arbes, Michelle Sever, Ben Vaughn, Jigna Mehta, Jeffrey T. Lynch, Herman Mitchell, Jane A. Hoppin, Harvey L. Spencer, Dale P. Sandler, Darryl C. Zeldin

**Affiliations:** ^1^Laboratory of Respiratory Biology, Division of Intramural Research, National Institute of Environmental Health Sciences, National Institutes of Health, Department of Health and Human Services, Research Triangle Park, North Carolina, USA; ^2^Rho, Inc., Chapel Hill, North Carolina, USA; ^3^Epidemiology Branch, Division of Intramural Research, National Institute of Environmental Health Sciences, National Institutes of Health, Department of Health and Human Services, Research Triangle Park, North Carolina, USA

**Keywords:** allergens, environment, epidemiology, sampling

## Abstract

Studies of indoor allergen exposures are often limited by the cost and logistics of sending technicians to homes to collect dust. In this study we evaluated the feasibility of having subjects collect their own dust samples. The objectives were to compare allergen concentrations between subject- and technician-collected samples and to examine the sample return rate. Using a dust collection device and written instructions provided to them by mail, 102 subjects collected a combined dust sample from a bed and bedroom floor. Later the same day, a technician collected a side-by-side sample. Dust samples were weighed and analyzed for the cat allergen Fel d 1 and the dust mite allergen Der p 1. Fifty additional subjects who were enrolled by telephone were mailed dust collection packages and asked to return a dust sample and questionnaire by mail. A technician did not visit their homes. Correlations between subject- and technician-collected samples were strong for concentrations of Fel d 1 (*r* = 0.88) and Der p 1 (*r* = 0.87). With allergen concentrations dichotomized at lower limits of detection and clinically relevant thresholds, agreements between methodologies ranged from 91 to 98%. Although dust weights were correlated (*r* = 0.48, *p* < 0.001), subjects collected lighter samples. Among the group of 50 subjects, 46 returned a dust sample and completed questionnaire. The median number of days to receive a sample was 15. With some limitations, subject-collected dust sampling appears to be a valid and practical option for epidemiologic and clinical studies that report allergen concentration as a measure of exposure.

Epidemiologic and clinical studies typically estimate indoor allergen exposures by analyzing samples of settled dust collected at one or more sites within the home (Platts-Mills et al. 1997; Pope et al. 1993). One of the major limitations of these studies is the need for a technician to make home visits. Because home visits are expensive in terms of labor costs and pose logistical challenges for studies conducted in multiple or distant geographic areas, researchers often conduct studies with fewer subjects and with fewer repeated measurements than would be ideal. Undoubtedly, the limitations imposed by home visits is why only one national survey of indoor allergens has been conducted in the United States (Vojta et al. 2002).

One alternative would be to rely on questionnaire data alone to predict allergen levels; however, home characteristics “may not be sufficiently predictive for many clinical and epidemiologic purposes” (Chew et al. 1998). Another alternative would be to have study subjects collect and mail in their own dust samples. If the feasibility of such a methodology could be demonstrated, there would be many applications for its use, such as epidemiologic studies that examine the relationships between allergen exposures and disease, clinical studies in which repeated measurements of indoor allergens are required, and the national surveillance of indoor allergens.

The objective of this study was to evaluate the feasibility—in terms of validity and sample return rate—of having subjects collect their own dust samples. The methodology developed for this study was centered around a commercially available dust collection device that attaches easily to most vacuum-cleaner hoses. Validity was assessed by comparing allergen concentrations and dust weights between paired subject- and technician-collected dust samples. Sample return rate was assessed in another group of subjects who volunteered to collect a dust sample and return it by mail.

## Materials and Methods

### Study subjects.

This study was conducted in two phases. The recruitment goal was to enroll 100 subjects for phase I and 50 subjects for phase II. Eligibility criteria were the same for all subjects: reside within 50 miles of the National Institute of Environmental Health Sciences (NIEHS), be at least 21 years of age, speak and read English, and have access to a vacuum cleaner with an extension hose. Subjects, who were recruited through flyers posted at shopping centers, apartment complexes, and convenience stores, were enrolled by telephone. For their participation in the study, subjects were offered a gift certificate valued at $50. Subjects were informed at enrollment that study results would not be reported to them. The study, which was conducted from June 2003 to January 2004, was approved by the NIEHS Institutional Review Board.

### Phase I.

**Figure 1:**
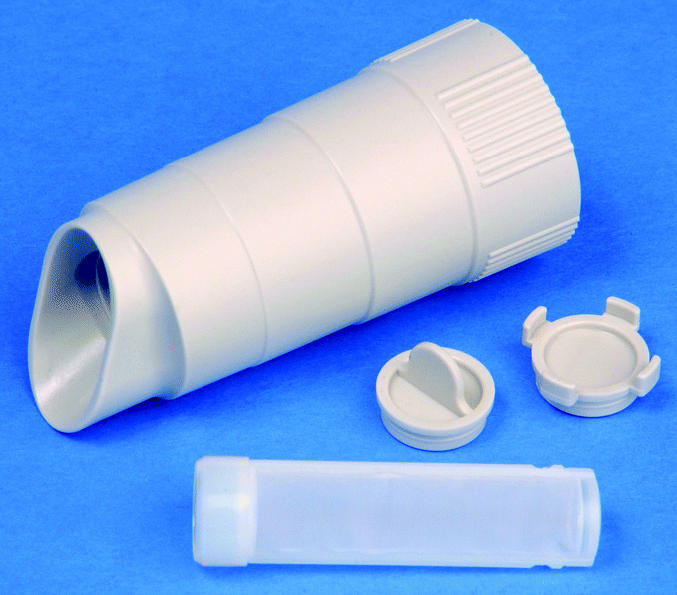
Example of a Mitest dust collector (Indoor Biotechnologies Inc.), along with its lids and dust filter, which subjects attached to their vacuums to collect a dust sample.

The purpose of phase I was to quantitatively compare pairs of side-by-side dust samples, with one of each pair collected by the subject and the other by a trained technician. Subjects were mailed a dust collection package containing an introductory letter, a dust collection instruction card, a Mitest dust collector (Indoor Biotechnologies Inc., Charlottesville, VA), two 18 × 24 inch measuring templates, a self-administered questionnaire, and a postage-paid, pread-dressed mailing envelope. The Mitest dust collector is a commercially available plastic device that fits on the distal end of most vacuum cleaner hoses (Figure 1). The collector contains a 40-μm nylon mesh filter that traps vacuumed dust. Using the 10-step procedure described on the dust collection instruction card (Table 1; the instruction card also included illustrations), subjects collected a combined bed and bedroom floor sample.

On the same day, but after the subject had collected the sample, a technician visited the subject’s home and asked the subject to identify the areas sampled. Arrangements for this visit were made during the enrollment telephone call. The technician collected a sample adjacent to, but not overlapping, the area reportedly sampled by the subject. The technicians used the same protocol the subjects used, with three exceptions. First, the technicians used a Eureka Mighty-Mite, model 3685, type B vacuum cleaner for all sample collections (Eureka Company, Bloomington, IL). Second, technicians used the dust collection device employed in the National Survey of Lead and Allergens in Housing rather than the Mitest Dust Collector (Vojta et al. 2002). This device consisted of a 19 mm × 90 mm cellulose extraction thimble (Whatman International Ltd., Maidstone, UK) placed in the distal end of the vacuum’s extension tube and covered with a clean crevice tool. A rubber O-ring placed around the thimble created a seal between the thimble and the vacuum tube. Third, technicians hand-delivered dust samples to the NIEHS, whereas subjects mailed in their samples. Five NIEHS-trained technicians, using three Mighty-Mite vacuum cleaners, collected dust samples in phase I.

### Phase II.

The purpose of phase II was to examine the sample return rate among a group of subjects who were enrolled by telephone and mailed a dust collection package. The introduction letter instructed the subjects to follow the steps on the dust collection instruction card, as described in Table 1. Study staff recorded the dates that dust collection packages were mailed out and the dates that dust samples were received at the NIEHS. A formal procedure for reminding subjects to return a sample was not written into the protocol. In general, the study manager telephoned a subject if he or she did not return a sample within a month.

### Laboratory procedures.

The processing and analyses of the dust samples followed protocols used in the National Survey of Lead and Allergens in Housing (Vojta et al. 2002). In the NIEHS laboratory, subject- and technician-collected samples were stored at −20°C until processing. Phase I and II dust samples were sieved through 425-μm-pore grating and weighed. Phase I dust samples were extracted in phosphate-buffered saline and clarified by centrifugation. Supernatants were decanted and stored at −20°C. Concentrations of the cat allergen Fel d 1 and the dust mite allergen Der p 1 were measured with monoclonal-antibody–based, enzyme-linked immunosorbent assays (Chapman et al. 1987; de Blay et al. 1991). Subject- and technician-collected samples from the same home were always paired on the same microtiter plate. The lower limits of detection were 0.0032 μg allergen/g of dust for Fel d 1 and 0.010 μg/g for Der p 1. For statistical analyses, samples below the limit of detection were assigned the value of 0.5 times the lower limit of detection.

### Statistical analyses.

All analyses, with the exception of calculating mean coefficients of variation, were conducted on the log_10_-transformed values of allergen concentrations and dust weights to stabilize variances. Paired *t*-tests were conducted to test whether the average difference between pairs of log-transformed values was different from zero. Pearson’s correlation coefficients and mean coefficients of variation that compared data between the two methods were calculated. Mean coefficients of variation were calculated from linear models that regressed the standard deviation of each data pair against the mean of each data pair. Regression equations that compared allergen concentrations and dust weights between methods were estimated using generalized linear models with technician values as the independent variable and subject values as the dependent variable. For comparisons between demographic groups, separate slopes were fit for each group and tested against each other. All analyses were conducted using SAS statistical software (SAS release 8.2; SAS Institute Inc., Cary, NC).

Of the 102 subjects enrolled in phase I, one subject collected a sample from a living room floor rather than a bed and bedroom floor and was dropped from statistical analyses. Although all subject-collected samples in phase I contained dust, two samples had only trace amounts of dust remaining after sieving, so those two samples could be neither weighed nor analyzed for allergens. However, for statistical comparisons of sample weights, those two samples were given the value of 0.0005 g (0.5 times the scale’s precision). Thus, 101 paired observations were available for statistical comparisons of dust weight, and 99 were available for statistical comparisons of Fel d 1 concentrations. Eleven samples with low dust weights were consumed in Fel d 1 laboratory analyses, which were given priority over Der p 1 laboratory analyses. Therefore, a total of 88 paired observations were available for Der p 1 statistical analyses.

## Results

### Characteristics of subjects.

The demographic characteristics of the 102 subjects enrolled in phase I and the 50 subjects enrolled in phase II are shown in Table 2. In each phase, most subjects were female. Almost all subjects were either white or black, with each of these racial groups being well represented in each phase. Only two phase I subjects and no phase II subjects were Hispanic. Approximately one-half of the subjects in each phase had college degrees. The average ages of phase I and II subjects, respectively, were 36.4 years and 39.0 years, with a wide range of ages represented.

### Comparison of Fel d 1 concentrations.

**Figure 2:**
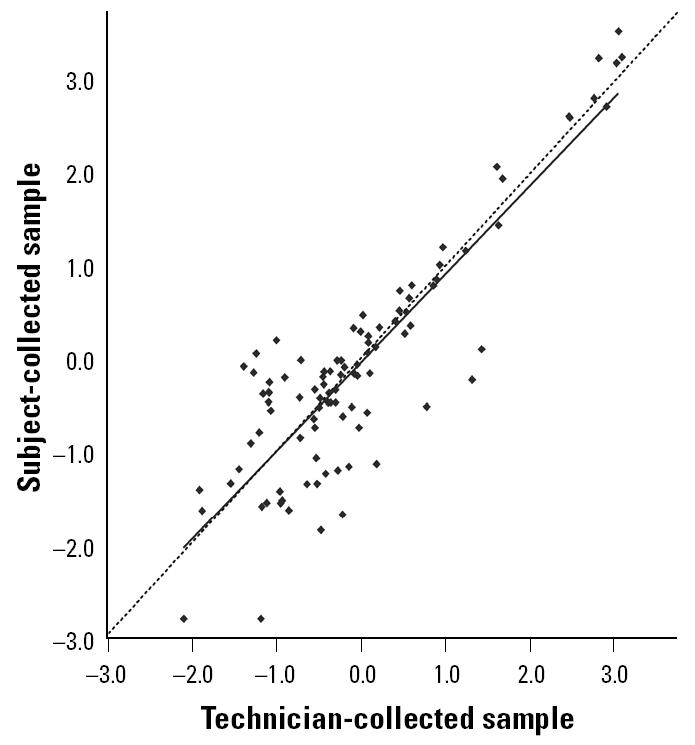
Scatter plot and regression line for the comparison of log-transformed Fel d 1 concentrations (μg/g) between subject- and technician-collected samples. The dashed line is the reference line, which has a slope of 1.0. [*n* = 99; *r* ^2^ = 0.77; *y* = −0.03 + 0.95(*x*)].

The geometric mean concentrations (micrograms per gram) of Fel d 1 for subject- and technician-collected samples were 0.87 ± 0.251 (SE) and 0.94 ± 0.249, respectively. The average difference between paired values was not different from zero (*p* = 0.598). The correlation between subject- and technician-collected samples was very strong (*r* = 0.880, *p* < 0.001). As shown in the scatter plot in Figure 2, the regression line, with a slope of 0.95, is essentially undistinguishable from the reference line, which has a slope of 1.00. The mean coefficient of variation, which was calculated on the untransformed data, was 52 ± 2.2% (SE).

When Fel d 1 concentrations were dichotomized at the lower limit of detection (0.0032 μg/g) and the proposed thresholds for allergic sensitization (1 μg/g) and asthma symptoms among allergic patients (8 μg/g), agreements between subject- and technician-collected samples were 98.0, 90.9, and 98.0%, respectively (Custovic et al. 1998; Gelber et al. 1993).

### Comparison of Der p 1 concentrations.

**Figure 3:**
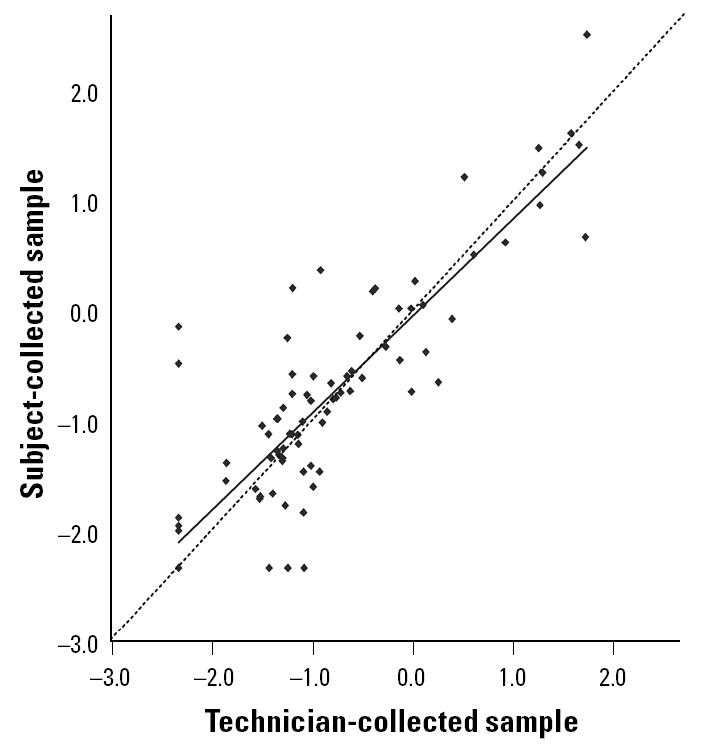
Scatter plot and regression line for the comparison of log-transformed Der p 1 concentrations (μg/g) between subject- and technician-collected samples [*n* = 88; *r*^2^ = 0.75; *y* = −0.04 + 0.88(*x*)]. The dashed line is the reference line, which has a slope of 1.0.

The geometric mean concentrations (micrograms per gram) of Der p 1 for subject- and technician-collected samples were 0.15 ± 0.039 (SE) and 0.13 ± 0.033, respectively. As with Fel d 1, the average difference in paired concentrations was not different from zero (*p* = 0.258), and the correlation between methodologies was very high (*r* = 0.868, *p* < 0.001). The regression line, with a slope of 0.88, closely approximates the reference line (Figure 3). The mean coefficient of variation was 93 ± 2.7%.

When Der p 1 concentrations were dichotomized at the lower limit of detection (0.010 μg/g) at the proposed thresholds for allergic sensitization (2 μg/g) and asthma symptoms among allergic individuals (10 μg/g), agreements between subject- and technician-collected samples were 90.9, 96.6, and 96.6%, respectively (Korsgaard 1998; Kuehr et al. 1994; Lau et al. 1989; Platts-Mills et al. 1987; Sporik et al. 1990).

### Comparison of dust weights.

**Figure 4:**
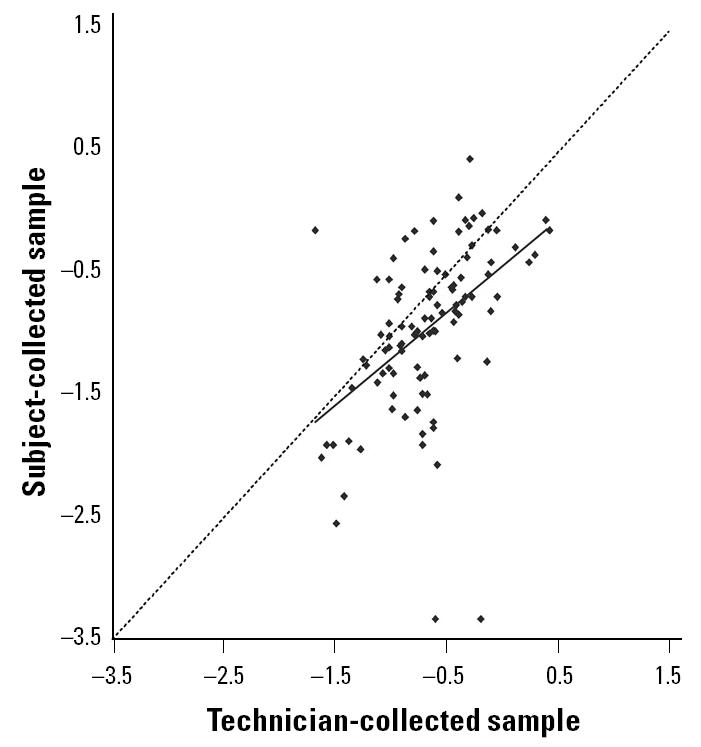
Scatter plot and regression line for the comparison of log-transformed dust weights (g) between subject- and technician-collected samples [*n* = 101; *r* ^2^ = 0.23; *y* = −0.44 + 0.76(*x*)]. The dashed line is the reference line, which has a slope of 1.0.

The correlation in dust weights between the two collection methods was significant (*r* = 0.481, *p* < 0.001); however, subjected-collected samples were lighter than technician-collected samples (paired *t*-test, *p* < 0.001). The geometric mean dust weights of subject- and technician-collected samples were 0.116 ± 0.018 g (SE) and 0.224 ± 0.022 g, respectively. The scatter plot and regression line in Figure 4 illustrate the tendency for subjects to collect lighter samples. Further analyses indicated that the slope of the regression line was not significantly modified by sex (*p* = 0.383), race (white vs. nonwhite, *p* = 0.562), education (any college vs. no college, *p* = 0.218), age (above median vs. at or below median, *p* = 0.200), size of bed (king or queen vs. smaller, *p* = 0.990), or whether the bed sheets had been washed within the previous 5 days (yes vs. no, *p* = 0.813). Although few hard-surfaced floors were sampled, the slope of the regression line was significantly modified by the type of floor surface (*p* = 0.004), with weights of the subject- and technician-collected samples being more similar if the floor was hard surfaced (slope = 1.16 ± 0.156; SE) rather than carpeted (slope = 0.69 ± 0.116). The slope of the regression line was also modified by the recency of cleaning the floor (*p* = 0.058), with weights being more similar between methods if the floor had not been cleaned within 5 days (slope = 0.94 ± 0.122) rather than within 5 days (slope = 0.71 ± 0.128). The mean coefficient of variation for the dust weight comparison was 66 ± 2.9%.

### Sample return rate.

Of the 50 subjects who were enrolled in phase II, 46 returned a dust sample and a completed questionnaire by mail, giving a return rate of 92%. The average number of days for the NIEHS to receive a dust sample from a subject was 19.6 ± 2.28 (SE). The minimum, median, and maximum were 6, 15, and 88 days. With the outlier of 88 days excluded from the analysis, it took males longer on average than females to return a sample (22.8 vs. 15.7 days, *p* = 0.053); however, there were no significant differences by race (*p* = 0.217), age (*p* = 0.679), or education (*p* = 0.317). Demographic information was not available on the four subjects who did not return a questionnaire, but even had this information been available, there were too few subjects in this group to characterize.

The geometric mean dust weight of the phase II samples was 0.144 ± 0.041 g. Two samples had insufficient amounts of dust to weigh. Geometric mean dust weights for subject-collected samples did not significantly differ between phases I and II (two-sample *t*-test *p* = 0.468).

## Discussion

This study provides evidence that subject-based sampling would be a valid option for measurements of indoor allergen concentrations. Correlations between subject- and technician-collected samples were very strong for concentrations of the two allergens tested, and percent agreements between the methodologies were very high when concentrations were categorized above and below clinically relevant thresholds. In addition, there was no evidence of systematic bias for comparisons of allergen concentrations, as evidenced by the regression slopes. Because the distributions of the untransformed concentrations and dust weights were highly skewed, as is the case in most allergen studies, mean coefficients of variation on the original scale were naturally high.

The correlation coefficients in this study were higher than those reported in a study that compared paired samples collected by a technician using the same vacuum cleaner (Wickens et al. 2004). In that study, which evaluated two somewhat similar dust collection devices, a technician collected side-by-side samples from longitudinal halves of 37 mattresses and duplicate samples from 37 floors (the sampled floor area was vacuumed twice) (Wickens et al. 2004). Pearson’s correlation coefficients for concentrations of Fel d 1 and Der p 1, respectively, were 0.76 and 0.67 for the bed and 0.82 and 0.58 for the floor (Wickens et al. 2004). Other studies have indicated that allergen concentrations vary between pairs of side-by-side bed and floor samples (Hirsch et al. 1998; Marks et al. 1995). This variation is due to random error, variation in laboratory assays, and the heterogeneity of allergen concentrations across surfaces.

For dust weight, subjects tended to collect lighter samples. One potential explanation is that subjects might not have been as thorough as the technicians in vacuuming the entire area within the template, especially when the floor surface was carpeted. We have observed that it is easier to move the dust collection device (regardless of type) across a hard surface than a carpeted surface, which may explain why dust weights were more similar when the sampled floor was hard surfaced. A second potential explanation is the difference in vacuum cleaners used by the subjects and the technicians. Technicians used the same make and model of vacuum cleaner throughout the study, and each of the three vacuum cleaners was new at the start of the study and had clean dust bags. Subjects used their own vacuum cleaners, which on average might have been less efficient than those used by the technicians. A third potential explanation is that the Mitest dust collector used by the subjects is less efficient in collecting dust than is the thimble device used by the technicians. The thimble device was used by the technicians because at the time of this study, we considered it to be the gold standard. However, our own side-by-side testing of these devices on six beds, eight carpeted floors, and six hard-surfaced floors did not reveal a significant difference in log-transformed dust weights across these 20 paired samples (data not shown; paired *t*-test, *p* = 0.475).

Whether differences in sample dust weights would affect the results of a study depends on whether the reported measure of exposure is allergen concentration or load. Studies typically report concentration (micrograms of allergen per gram of dust), which theoretically would be the same regardless of the amount of dust collected. However, for studies reporting allergen load, which is the product of the sample dust weight and allergen concentration, lower dust weights would result in lower allergen loads. Because the estimation of allergen load is very sensitive to the efficiencies of the vacuuming equipment and the dust collection device and to variations in vacuuming technique, we question, as have others (Wickens et al. 2004), whether load is a reliable measure of allergen exposure.

The major limitation to this subject-based sampling is that it requires subjects (or someone in their household) to have access to a vacuum cleaner, to be able to read and follow written instructions, and to be physically capable of completing the sampling procedure. Vacuum cleaner ownership is not universal, nor is literacy, and it is not likely that all subjects in a target population would be physically capable of performing the procedure. Therefore, this methodology would not be feasible for every study, especially for a study that targeted low-income, inner-city households or the very elderly. In epidemiologic surveys limited only to subjects who could carry out the procedure, people of low economic status and the very elderly might be underrepresented, which could reduce the generalizability of the results. The exclusion of these groups in clinical and case–control studies could also reduce the generalizability of results, although it would not be a source of bias as long as intervention (case) and control groups were limited by the same inclusion criteria. Depending on the study size and the number of subjects who could not perform the procedure themselves (or have a household member perform the procedure for them), researchers could combine subject- and technician-based sampling within the same study because the two methods give very similar results. Also, if a relatively small number of subjects did not have vacuum cleaners, the study could provide vacuum cleaners to those subjects. In fact, for small studies in which repeated sampling is needed through time, it would be more economical to provide vacuum cleaners to all subjects than to have technicians make repeated home visits. This would be especially true for studies that send out pairs of technicians to ensure their safety, as was the case in the National Cooperative Inner-City Asthma Study (Mitchell et al. 1997).

The sample return rate among the 50 phase II subjects who volunteered to collect a dust sample and return it by mail was quite good, especially when one considers that a formal call-back procedure was not in place. In general, the study manager telephoned a subject if his or her sample was not received at the NIEHS within a month. Among the 46 subjects who returned a sample and a completed questionnaire, only four were reminded by telephone, and each of them was contacted only once. The subjects who did not return a dust sample were contacted two or three times. Also, because allergen results were not reported to subjects, there was no direct benefit to subjects for their participation, other than the $50 they received. In studies of asthmatic or allergic subjects in which allergen levels would be reported to subjects, one might expect them to be even more motivated to comply with the dust collection protocol. It should be noted that phase II measured sample return rate as opposed to response rate. The subjects in phase II responded to advertising and agreed to participate in the study before the dust collection packages were mailed out, as would be the case in most clinical trials and environmental intervention studies. If the dust collection packages had been sent out to a random sample of the population without enrolling them beforehand, as is typically done in surveys, the response rate would likely have been much lower than the sample return rate reported in this study. In addition, this study did not test what the response rate or quality of the samples would have been if subjects had been asked to collect multiple samples over time, as would be the case in longitudinal studies, which typically experience some loss to follow-up.

One of the major benefits of having subjects collect their own samples is lower cost. We estimated a cost of $18 per dust collection package (Mitest dust collector, printed materials, templates, mailing envelopes, and postage). For technician-based sampling, there would typically be those same costs (technicians are often required to ship samples) plus the cost of training, equipment, labor, and travel. Even for a local study, the cost of having a technician collect a sample would be $75–100.

In conclusion, subject-collected dust sampling appears to be a valid method for measuring allergen concentrations in homes, and although it has some limitations, it should be considered a feasible option for many epidemiologic and clinical studies.

## Figures and Tables

**Table 1:** The 10-step procedure described on the dust collection instruction card.

Bring to your bedroom: the dust collector and its cap, the two measuring squares, a watch or clock with second hand, and your vacuum cleaner. Roll back the covers on your bed. Place one square on the bottom or fitted sheet. Place the other square on the floor beside the bed. Place the dust collector on the end of your vacuum hose (it may fit loosely until the vacuum is turned on). Prepare your watch or clock for timing. Vacuum the area within one square for exactly 2 min. Without turning the vacuum off, continue to step 5. Vacuum the area within the other square for exactly 2 min. While holding the collector up, turn the vacuum off. Push the cap firmly into the top of the collector. Remove the collector from the hose and place it back into the Ziploc bag and close. Complete the questionnaire. Place the following items in the return mailing envelop: the Ziploc bag containing the dust collector and the completed questionnaire. Place the return envelope in the U.S. mail within 12 hr.

The card given to subjects also included illustrations.

**Table 2:** Characteristics (frequencies) of subjects enrolled in phases I and II.

Characteristics	Phase I (*n* = 102)	Phase II (*n* = 50)
Demographic characteristics		
Sex		
Female	81	31
Male	21	15
Unknown	0	4
Race		
White	55	21
Black	38	22
Asian	5	1
Native American	2	1
Pacific Islander	0	1
Unknown	2	4
Hispanic ethnicity		
No	100	45
Yes	2	0
Unknown	0	5
Education		
Some high school	3	3
High school	18	10
Some college	27	8
College degree	53	25
Unknown	1	4
Age (years)		
Mean ± SE	36.4 ± 1.22	39.0 ± 1.69
Median	33.5	37.5
Minimum	17	23
Maximum	75	71
Sample-related characteristics		
Type of vacuum used		
Upright	75	35
Canister	10	7
Small hand-held	9	3
Central	1	0
Other	4	1
Unknown	3	4
Bed sampled		
Twin/single	18	2
Double/full	31	11
Queen	39	19
King	13	13
Unknown	1	5
Floor covering sampled		
Carpet	93	44
Hard surface	7	2
Carpet and hard surface	1	0
Unknown	1	4
Changed sheets within 5 days		
No	60	35
Yes	41	9
Unknown	1	6
Cleaned floor within 5 days		
No	53	33
Yes	48	13
Unknown	1	4
